# “The Yak”—A remarkable animal living in a harsh environment: An overview of its feeding, growth, production performance, and contribution to food security

**DOI:** 10.3389/fvets.2023.1086985

**Published:** 2023-02-02

**Authors:** Ali Mujtaba Shah, Iqra Bano, Izhar Hyder Qazi, Maharach Matra, Metha Wanapat

**Affiliations:** ^1^Tropical Feed Resources Research and Development Center (TROFREC), Department of Animal Science, Faculty of Agriculture, Khon Kaen University, Khon Kaen, Thailand; ^2^Department of Livestock Production, Shaheed Benazir Bhutto University of Veterinary and Animal Sciences, Sakrand, Sindh, Pakistan; ^3^Department of Veterinary Physiology and Biochemistry, Shaheed Benazir Bhutto University of Veterinary and Animal Sciences, Sakrand, Sindh, Pakistan; ^4^Department of Veterinary Anatomy, Histology, and Embryology, Shaheed Benazir Bhutto University of Veterinary and Animal Sciences, Sakrand, Sindh, Pakistan

**Keywords:** yaks (*Bos grunniens*), environmental stress, growth retardation, climate change, domestication, feeding, microbiota, breeding

## Abstract

Yaks play an important role in the livelihood of the people of the Qinghai-Tibet Plateau (QTP) and contribute significantly to the economy of the different countries in the region. Yaks are commonly raised at high altitudes of ~ 3,000–5,400 m above sea level. They provide many important products, namely, milk, meat, fur, and manure, as well as social status, etc. Yaks were domesticated from wild yaks and are present in the remote mountains of the QTP region. In the summer season, when a higher quantity of pasture is available in the mountain region, yaks use their long tongues to graze the pasture and spend ~ 30–80% of their daytime grazing. The remaining time is spent walking, resting, and doing other activities. In the winter season, due to heavy snowfall in the mountains, pasture is scarce, and yaks face feeding issues due to pasture scarcity. Hence, the normal body weight of yaks is affected and growth retardation occurs, which consequently affects their production performance. In this review article, we have discussed the domestication of yaks, the feeding pattern of yaks, the difference between the normal and growth-retarded yaks, and also their microbial community and their influences. In addition, blood biochemistry, the compositions of the yaks' milk and meat, and reproduction are reported herein. Evidence suggested that yaks play an important role in the daily life of the people living on the QTP, who consume milk, meat, fur, use manure for fuel and land fertilizer purposes, and use the animals for transportation. Yaks' close association with the people's well-being and livelihood has been significant.

## 1. Introduction

The QTP is a region where the majority of yaks (*Bos grunniens*) are concentrated. Yaks are very old bovines that live at an altitude ranging from 3,000 to 5,400m with low atmospheric oxygen pressure. Yaks play a critical role in the lives of the local population and also the ecological niche in the ecosystem of the Plateau (1). People living in the QTP obtain milk, meat, fuel, and fur from yaks. Yaks are endothermic animals that can tolerate impaired oxygen supply. Normally, these yaks are semi-domesticated grazing animals, while natural breeding takes place and no formulated feed is commonly provided for them (2). Besides production, including meat and milk, yaks are also used for transportation purposes. The meat of yaks contribute significantly to the economy of the local people and the meat is popular among the people living in the QTP (3, 4).

The environment of the QTP is very harsh, particularly in the cold season, with a long snowfall season from October to May (Temperature −15 to −05). In the QTP, these months of long snowfall and cold weather cause a shortage of grass, and the grass is covered with a thick layer of snow, which affects the survival of these yaks (5). In addition, this shortage of grass during the winter season also causes malnutrition and retarded growth in yaks. In the cold season, the birth rate of calves is at its peak. Yaks normally conceive during late summer and give birth in the early summer (6). Due to a shortage of green fodder in harsh weather, calves face growth retardation (5). Hence, in the QTP, growth-retarded yaks are a common issue each year. Yaks are the world's most notable animals living and reproducing freely in such harsh plateau conditions. In addition, early nomadic people domesticated yaks more than 7,300 years ago and yaks are the only major species of livestock that coexist in the same ecosystem as their wild forebears (7). Among all livestock animals, yaks are the only domesticated animals that can survive and adjust to the cold and harsh environment of the QTP and face high food scarcity (8).

Earlier studies showed that malnutrition during the early life of animals damages the gastrointestinal tract function and structure, affecting the normal growth of the animals (2, 9). The use of different feed additives in the fodder of growth-retarded yaks can improve the structure and function of the gastrointestinal tract of the animals because the health of the gastrointestinal tract is important for the proper absorption of nutrients from the tract and to increase the growth of the animals (10). The bacterial community of the gastrointestinal tract also plays a crucial role in the growth and production performance of the animals (11). In addition, a stable bacterial community is crucial for the host to maintain the health and internal environment of the gastrointestinal tract (12).

## 2. Yak domestication

**Figure 1:**
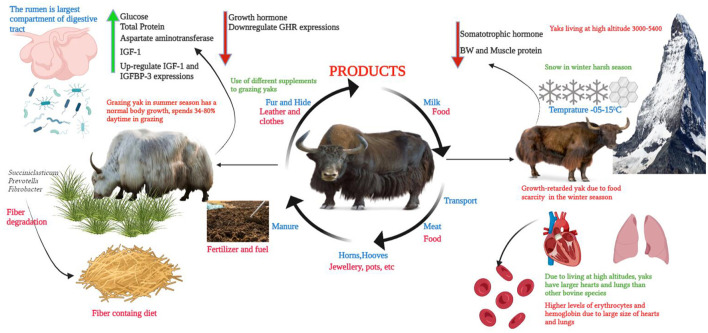
A brief diagram of the domestication, feeding, microbiota, and harsh environment of the QTP.

Yaks play a crucial role in the life of the people of Tibet, as they not only provide milk and meat but also hide, fur, and manure for land fertilization. In addition, the fur of yaks is used in the preparation of the local black tents that retain moisture when it rains, preventing tents from leaking. The long horns of yaks are used for the preparation of milk pails and other materials (13). The manure of yaks is not only used as a fertilizer but is also used as a construction material in walls, enclosures, and other different types of houses, as well as in the preparation of toys. It is observed that yaks are commonly crossbred with cattle to produce F1 hybrid dzo, which is a higher producer of milk compared to yaks and can survive in high altitudes (6). Yaks have the ability to adapt to the harsh environment of the winter season and live at high altitudes on the Plateau (Figure 1). The hide of yaks is very thick and helps the yak to protect its body from the cold and to keep it warm. Anatomically, its greater heart and lung size and the higher foraging ability of its adapted tongue allows the yak to eat the low-lying grasses easily in the area of the Plateau (14). The yak's genetic makeup allows for a unique ability to adapt the low-oxygen conditions of the QTP as compared to cattle (14).

Yaks are domesticated in different countries, such as Pakistan, Nepal, China, India, Kyrgyzstan, Russia, Tajikistan, Afghanistan, Bhutan, and Mongolia (Joshi), consisting of northern and southern zones connected in the west by the Pamir mountains. The entire QTP, east of Sichuan until Baltistan in the west, the Himalayas in Nepal, Bhutan, and India fall in the southern zone (15). This region shows the maximum range of the Tibetosphere, mostly occupied by speakers of Tibetic languages (16), but also speakers of Indo-Iranian (Indo-European), Burushaski (an isolated language), Mongolic, Turkic, and Sino-Tibetan languages that are culturally and linguistically influenced by Tibetan languages. Inconveniently, there is little research and literature available on the domestication of yaks in the archaeological department. In the Nuomohong sites on the northeastern Plateau, such as Xiariyamakebu and Tawendaliha (3,300–2,700 BP), physical evidence of yak skeletal remains was discovered; nevertheless, only their presence was reported, and it was unclear if they showed signs of domestication or even what domestication signs might look like in yaks. In Talitaliha (3,300–2,700 BP), a clay sculpture of a yak demonstrated the importance this animal may have held for the inhabitants of the site (13). In Qugong, a yak skull that dates to roughly 3,650 BC was also unearthed in an ashpit (17). The researchers contended that due to the horn's small size, it was probably from a domesticated yak or one hybridized with cattle. Yak skulls dating to 450 CE were also present in Samdzong 5 (18).

## 3. Yak feeding system

Yaks were domesticated from wild yaks (*Bos mutus*) and are present in the remote mountains of the QTP (19). Very little research was conducted regarding the feeding system of yaks (20, 21). Particularly in the summer season, when pasture covered with long grass is available in the mountain region, yaks use their long tongues for ingestion, while in short grass, they use lips and incisor teeth for ingestion (22) (Figure 1). In the winter season, when the pasture is fully covered with a big coat of snow, yaks use their hooves, horns, heads, and muzzles to remove the thick snow layers (23). This process helps the development of their sensory olfactory organs. However, in the scarcity period, yaks use the stem and leaves from residual wilted grasses and plants. In the winter season, yaks utilize more energy in grazing compared to the summer season. In summer, with the growth of new green herbage, the grazing time increases and even extends to midnight. Yaks spend more time idling in winter and early spring (March and April) since less herbage is available, and they spend more time searching or lying down to avoid the cold and strong winds (21). Growing and lactating yaks spend more time grazing because of their higher nutrient requirements. During snowfall and hail storms, yaks run over the pasture at very high speeds, of ~3,400 m/h (24). Yaks graze on alpine pastures, such as alfalfa, oak, and other herbages that are commonly available in mountain regions. Normally, yaks avoid grazing onpoisonous plants but in scarcity periods due to a shortage of pasture, they graze on poisonous weeds and suffer from poisoning. The use of different concentrates and urea molasses blocks is effective in improving the production of grazing yaks and maintaining body weight in winter. Yaks consume less feed compared to cattle due to their small rumen size and capacity (25). Yaks prefer fresh grass and high temperature affects their feed intakes. Yaks normally drink water from rivers and in the absence of water, particularly in the winter season, they lick ice to meet their water requirement (26). Ma et al. (27) concluded that the addition of glutamine in the diet of yaks improved the growth of slow-growing yaks, as wel l as gastrointestinal morphology and barrier function. In another study, study authors reported that the supplementation of the diet of grazing yaks with multi-nutrient blocks improved body weight gain and improved the health of yaks (28). Zhao et al. (29) reported that rumen-protected methionine (50 g/day) supplementation in the diet of lactating yaks increased the dry matter intake, milk production, milk protein content, and lactoferrin concentration. In addition, rumen protect methionine also improved the rumen fermentation characteristics in lactating yaks. The concentrate supplementation (CP 18%, 12.49 MJ ME/Kg) of grazing yaks during the warm season improves body weight gain and ruminal fermentation efficiency, mainly by an increase in MCP and VFA productions and by lowering acetate to propionate ratio. In addition, supplementation of concentrates reduced bacterial richness, diversity indices, and relative abundance of succiniclasticum, Prevotellaceae_UCG_003, Prevotel-laceae_UCG_005, and Ruminococcus_1, while they enhanced the relative abundance of the Christensenellaceae_R-7_group and the Ruminococca-ceae_NK4A214_group (30). In addition we have mentioned the effects of different feed supplements on the growth and production performance of yaks in Table 1.

**Table 1:** Effects of different feed supplements on the growth and production performance of yaks.

**Supplement**	**Trial duration**	**Dose**	**Substrate/feed**	**Key findings**	**Reference**
Rumen Protected Methionine		50 g/day	TMR	Increase DMI, Milk Yield, and Protein content	(29)
Inoculated Oat Silage	5 days		TMR	Increased feed intake, rumen fermentation, Milk Yield, and essential Amino Acid	(31)
Trace Minerals (iron (Fe), copper (Cu), manganese (Mn), zinc (Zn), cobalt (Co), iodine (I) and selenium (Se)			TMR	Increase Milk Yield, Milk protein lactoferrin concentrations. Altered rumen bacteria )	(32)
Multi-Nutrient Blocks		Grazing+150,250 and 500 g/day	TMR	Increase Body weight gain	(28)
Dolichousnea longissima (Ach.)	28 days	3 kg/day	TMR	Increase Protein content in milk, Maintain body condition score	(33)
High CP and ME levels in the diet	50 days		TMR	Increased daily weight gain	(34)
Astragalus root extract	60 days	5-8% DMI	TMR	Increased average daily gain, reduced feed to gain ration, Antioxidant capacity and immunity enhanced	(35)
Glutamine	90 days	180 g/day	TMR	Improved gastrointestinal morphology, reduction in the inflammatory response and improved gastrointestinal barrier function	(27)
Herbal roots *Codonopsis pilosula* (Chinese Medicinal plants)	60 days	80 mL/kg	Concentrate	Improved daily weight gain, improved gastrointestinal microbiota communities	(36)
Rumen-protected Lysine and Methionine	32 days	15 g/day, 5 g/day	TMR	Altered the composition of rumen microbiota and VFAs concentrations of lactating yaks	(37)

TMR, Total Mixed Ration; DMI, Dry matter intake.

## 4. Normal and growth-retarded yaks

The QTP environment is very harsh, particularly during the cold season due to high snowfall from October to May (Temperature −15 to −05). Due to this high snowfall and cold season, the shortage of grass occurs during these months, which affects the survival of yaks (38). In addition, this shortage of grass during the winter season also causes malnutrition and retarded growth in yaks (Figure 1). During the cold season, the birth rate of calves is at its peak, and due to a shortage of green fodder, calves face growth retardation (5). Therefore, at the QTP, growth-retarded yaks are a common issue each year. Usually, yak growth retardation is defined as the yak's body weight being twice the standard deviation below the average weight of a yak population of the same breed and age (39). During the early life span, severe malnutrition (pregnancy and neonatal periods) affects the growth of the animals and causes growth-retarded yaks. These growth-retarded yaks are commonly found in the QTP with lower body weight and high morbidity and mortality rates (38). Hu et al. (39) stated that the serum somatotropic hormone axis concentration is significantly reduced in growth-retarded yaks in comparison to normal yaks. Hu et al. (1) showed that the initial body weight of growth-retarded yaks was significantly lower than that of healthy yaks. Ma et al. (8) concluded that growth performance of the growth-retarded yaks was poor in contrast to normal yaks, and the average daily gain and final body weight of growth-retarded yaks were differently lower than the control group. Dai et al. (30) demonstrated that supplementation improved the body weight of yaks compared to grazing-only yaks and that supplementation also increased the average daily gain of these yaks when compared to grazing-only yaks.

## 5. Gut microbial community in growth-retarded and normal yaks

The microbiota in the gut plays an important role in the digestion of food and absorption of nutrients from the diet, in regulating the storage of fat, in stimulating intestinal epithelial renewal, and in directing immune system maturation (40). Ma et al. (2) reported that in the rumen and colon of normal yaks and growth-retarded yaks the alpha-diversity analysis showed that the observed species, *Chao1, ACE*, and Shannon indexes were similar between groups. The Shannon index in the duodenum and ileum of the growth-normal yak group was higher when compared to that of the growth-retarded yak group. However, in the jejunum and cecum, an opposite trend was observed. In the duodenum and ileum, no significant difference was found in species *Chao1*, and in *ACE* indexes in either group. Nonetheless, in the jejunum and cecum of growth-normal yaks, the observed species *Chao1, and ACE* indexes were lower compared to the growth-retarded yaks. In addition, the Bray–Curtis dissimilarity matrices, when using the PCoA to examine the structure of microflora in the GIT between the growth-normal yak and growth-retarded yak groups, and the bacterial communities in the rumen, duodenum, jejunum, ileum, and cecum were separated from each other (2).

It was noted that out of 32 phyla from 12 rumen samples, the number of bacteria phyla found in normal-growth and retarded-growth yaks was 29 and 31, respectively. In the rumen of the yaks, *Firmicutes* and *Bacteroidetes* were the predominant bacteria, accounts for at 75% of the total relative abundance of all bacteria. The relative abundance of the *Spirochetes* was higher in normal-growth yaks when compared to retarded-growth yaks. However, *Actinobacteria* and *Patescibacteria* relative abundance was found to be higher in the retarded-growth yaks than in the normal-growth yaks. In addition, *Firmicutes* and *Tenericutes* relative abundance were found to be higher in normal-growth yaks than in retarded-growth yaks. However, *Chloroflexi* relative abundance showed an opposite trend between the two groups. No significant difference in the *Firmicutes*-to-*Bacteroidetes* ratio was found in the normal-growth and retarded-growth yaks. The level of genus of the *Rikenellaceae RC9* gut group was found to be the most dominant bacterium in the rumen digesta of the normal-growth and retarded-growth yaks, followed by *Bacteroidales unclassified F082, Christensenellaceae R-7* group, and *Prevotella 1*. The relative abundance of *Treponema 2, Ruminococcaceae UCG-014, and Ruminococcaceae NK4A214* were found to be higher in normal-growth yaks than in retarded-growth yaks. The relative abundance of the group of *Christensenellaceae R-7* was found to be higher in retarded-growth yaks than in normal-growth yaks. Furthermore, the relative abundance of the group of *Bacteroidales UCG-001*, was found to be slightly higher in normal-growth yaks than in retarded-growth yaks. LEfSe analysis further classified different genera according to the relative abundance of different bacteria. In the rumen of normal-growth and retarded-growth yaks, a group of 13 bacteria was found in *Ruminococcaceae UCG-014, Christensenellaceae R-7* group, and *Treponema 2*, respectively. Du et al. (41) conducted research on growth-retarded calves and supplemented their diet with the probiotic *Bacillus amyloliquefaciens C-1* and found that body weight, feed intake, and efficiency of feed conversion ratio of the growth-retarded calves were increased compared to the control group. Moreover, the abundance of bacteria contributing to the production of energy and saturated fatty acids (short-chain fatty acids), including *Proteobacteria, Rhodospiril-laceae, Campylobacterales*, and *Butyricimonas*, were increased in growth-retarded calves supplemented with probiotics compared with the control group, and the suspected pathogens, which included *Anaeroplasma* and *Acholeplasma* were decreased with probiotic supplementation.

## 6. Gut microbiota in yaks

In extremely harsh environments, feed selection and feed intake influence the survival and health of yaks. The microbiota plays a crucial role in the digestion of nutrients, particularly in fiber digestion and synthesis of microbial protein, and helps the host to adapt to the environment (42). Different researchers suggested that feed composition is the main driver altering the microbial population in the gut. In all mammals, gut microbiota constitutes a complex and crucial ecosystem. Recent studies showed that the gut microbiota in yaks plays an important role in adaptation to the harsh environment, namely the cold season, though the composition of the microbiota of the yak is poorly categorized (43). The rumen of the yak is the largest section among all the sections of the gastrointestinal tract and the microbiota in this section plays a crucial role in the digestion of fiber and non-fiber carbohydrates and characterizes the major site of fermentation in yaks.

Su et al. (44) stated that in the gastrointestinal tract of grazing yaks more than three taxa were identified at the Phylum level during the summer season. These phyla were *Firmicutes, Bacteroidetes*, and *Proteobacteria*. At the genus level, eight genera were identified in the gastrointestinal tract of the yaks, such as *Succiniclasticum* (45), *Prevotella* (46), and *Fibrobacter* (47). It has been frequently reported from fecal samples that the gut microbiota in yaks are *Christensenellaceae_R-7, Ruminococcaceae_UCG-005, Ruminococcaceae_NK4A-214, Rikenellaceae_RC9, Prevotellaceae_UCG-003*, and *Prevotellaceae_UCG-001*, which are implicated in fiber digestion. Moreover, *Akkermansia* and *Bacteroides* were associated with enhancing the host immune responses and metabolic functions (48), and *Ruminococcaceae_UCG-010* was reported as well. In this experiment, the subsequent downstream analysis selected these taxa as they appear to be the main representatives of gut microbiota in yaks. The rumen is the largest compartment in the yak digestive tract and it is in the rumen thatbacterial fermentation takes place and volatile fatty acids are produced. These volatile fatty acids are the main energy source for yaks. The rumen dominates the physiology of the animal, as well as the microbiota, and has a high concentration of *Firmicutes* and *Bacteriodetes*. In the four stomachs compartment, the genera *Rikenellaceae_RC9, Prevotella, Christensenellaceae_R-7, and Prevotellaceae_UCG- 001* were found to be most dominant. In the rumen, *Prevotella* was highly prevalent but its levels displayed a reducing trend toward the reticulum and abomasum (44).

Global warming is caused by greenhouse gases, especially methane gas, which is the main component that directly contributes to global warming. The yak is a local and indigenous animal that produces low methane in the environment when compared to other ruminants living at low altitudes, such as cattle and sheep (3). Xue et al. (49) reported that yak indoor feeding is a newly developing factor that can increase the production of bacteria that are involved in methane production in the gut. Hence, in this respect, using advanced feeding treatments to decrease the production of bacteria that are involved in methane production should receive more attention (44). In the intestine of ruminants, trillions of microbes are present that play an important role in the host's health, as well as in the metabolism, digestion, and absorption of nutrients in the body and homeostasis of the intestine in yaks (50). Furthermore, microbiota in the gut also plays an important function in the maturation of the immune system, permeability, and differentiation of the epithelium of the intestine (51). Statistically, in the healthy intestine, more than 1,014 microbes are available, about tenfold the total quantity of host cells (52). Amid them, bacteria in the intestine account for about 98% of the total population of the microbiota, while fungi (0.1%), viruses, and protozoa (53) comprise the rest. Gut microbiota in stable conditions is needed for numerous physiological and metabolic processes, while the destabilization in the microbiota community may result in different gastrointestinal problems, such as enteritis, diarrhea, and irritable bowel syndrome. Guo et al. (54) researched the microbiota of yaks and found that on 97% of the similarity level, a total of 7,200 OTUs were recognized, and among them, 6,642 OTUs were related to bacteria and only 40 OTUs belonged to archaea. At OTUs levels, a rarefaction test was subsequently conducted.

In addition, Guo et al. (54) reported that in the taxonomy summary of the rumen bacterial community, a total of 23 phyla were noted in the rumen. Among these phyla, the six most abundant phyla were *Firmicutes, Bacteroidetes, Proteobacteria, Spirochaetes, Tenericutes*, and Verrucomicrobia and the remaining phyla were in lower abundance. At the taxonomic level, a more detailed analysis was done and found that the richest abundance of *Proteobacteria* contained the six classes, whereas Firmicutes and Bacteroidetes only had three and four classes, respectively, though Bacteroidia and Clostridia were the two predominant classes. At the family level, the most abundant families were Unclassified Bacteroidales, Ruminococcaceae, Unclassified Clostridiales, Lachnospiraceae, BS11, and Prevotellaceae. Other families were also found but at lower percentages. Although these yaks did not have major clinical problems, the growth-retarded yaks faced greater mortality and eccentricity rates and poor feed utilization, which results in greater feed costs. The proper health of the gastrointestinal tract plays a crucial role in the growth performance of animals (55, 56).

Ma et al. (2) researched the microbiota of normal yaks and growth-retarded yaks and concluded that the yak gastrointestinal tract displayed diverse microbiota communities among the groups. In the gastrointestinal tract of the growth-normal yaks the relative abundance of bacteria, such as *Ruminococcaceae, Treponema, Clostridiaceae, Prevotellaceae, and Lachnospiraceae*, were higher compared to the growth-retarded yaks and these bacteria played a crucial role in the degradation of the oligosaccharide, starch, and cellulose. Furthermore, in the gastrointestinal tract of the growth-retarded yaks the relative abundance of bacteria, such as *(Eubacterium) tenue, Christensenellaceae R-7* group, and unclassified *Chitinophagaceae*, were higher compared to normal yaks. Ma et al. (2) stated that in the gastrointestinal tract of growth-retarded yaks the bacterial community was spared and that regulating the microbial population might be an effective solution to compensate growth of growth-retarded yaks. Xue et al. (57) reported that enteric methane is produced due to the degradation of the plant material by gastrointestinal microbiota in the rumen. In the yak, high throughput sequence data revealed that yak's rumen have highly diverse gastrointestinal microbial communities of unclassified microorganisms. The yak's prokaryotic community remains uncharacterized when compared with other livestock species. In addition, in yaks, the rumen's prokaryotic community consisted of 29 phyla, 40 classes, 63 orders, 77 families, and 79 genera. *Bacteroidetes* (59.1%) was the most abundant phylum, followed by *Firmicutes, Proteobacteria, Fibrobacteres*, and *Euryarchaeota*. *Prevotella* was the predominant genus, averaging 28.5% of all the rumen's prokaryotic genera. Archaea accounted for 2.26% of the total prokaryotic community, with their community dominated by Methanobacteriaceae (82%), followed by *Methanomassiliicoccaceae*, and Methanosarcinaceae. Huang et al. (58) investigated the methanogen diversity in yak and cattle by 16S rRNA gene sequences in the rumen and concluded that yaks have a unique ecosystem of microbiota in the rumen that is significantly changed from cattle. This helps explain why yaks produce less methane compared to cattle. In cattle's rumen, billions of microorganisms are present, such as bacteria, methanogenic archaea, protozoa, and fungi. The methanogen genera commonly found in the rumen of cattle are *Methanobrevibacter, Methanomicrobium, Methanobacterium*, and *Methanosarcina* (59, 60).

## 7. Blood biochemistry and hormones level in yaks

The level of glucose in serum reflects the utilization of energy in ruminants. When the level of serum glucose reduces, it indicates a lack of dietary energy or poor utilization of energy (30, 61). Mellado et al. (62) revealed that in goats the diet containing concentrate influenced the glucose concentration. This is because a diet containing a higher concentration of concentrate (350 g/day) contains more energy supply compared to a diet with a lower concentrate. In addition, in normal and healthy conditions, the animal's diet containing higher levels of protein increased the concentration of total protein in the blood (63). Unsurprisingly, diets containing supplements significantly enhanced the levels of total protein in yaks, mainly albumin and globulin (64). Chen et al. (65) showed that the aspartate aminotransferase level in serum improved with the provision of a diet with a high level of concentrate in the case of housing-feeding yaks. Dai et al. (30) stated that the use of different supplements in the diet of grazing yaks enhanced the levels of aspartate aminotransferase, glucose, and total protein as compared to grazing-only yaks. Though, no change was found in triglycerides, urea nitrogen, and alkaline phosphate concentrations between grazing-only and grazing yaks with supplementation. The higher altitude, cold stress, and forage scarcity changed the blood composition of the yaks compared to other bovine species, such as buffalo, cow, goat, and sheep (66). Due to high altitude, yak face chronic hypoxia and this stress affects the performance of the respiratory and cardiovascular systems in yaks. Due to this reason, the yaks have a larger pulmonary alveolar area per unit of time, thinner alveolar septum, and thinner blood-air barrier. In addition, yaks have larger lungs and hearts and greater levels of erythrocytes and hemoglobin compared to other bovine species (Figure 1) (66). Yang et al. (67) concluded that the provision of a higher energy diet for yaks increased the IGF-1 level and relative expressions of IGF-1 and IGFBP-3 and reduced the growth hormone levels and GHR expressions. In addition, seven days of starvation reduced the glucose, triglycerides, and blood urea nitrogen levels in yaks. However, NEFA and BHBA concentrations were fivefold higher in the starvation group compared to control groups, and after seven days of feeding, all of these parameters were found to be normal in the control group (46). Zhou et al. (68) reported that different concentrations of supplements for grazing yak enhanced the levels of albumin, blood urea nitrogen, and total protein compared to the control group. Gao et al. (69) stated that nutritional deficiency in the diet of grazing yaks had a bigger effect than the cold stress during the cold season. Meanwhile, an analysis of the random forest method showed that in the serum of grazing yaks, the metabolic profile related to the metabolism of amino acid and protein was altered and it was affected by nutrient deficiency than by the energy and minerals metabolism in cold conditions. Diet-containing supplements would be beneficial for grazing yaks in cold conditions.

## 8. Reproduction and breeding in yaks

Yaks are seasonal breeders like other bovine species and their mating and conception depend, annually, on the temperate season (70). Normally, yaks produce four to five calves during their entire life and they mature at the age of 3–4 years old (71). Various studies showed that different environmental conditions affect the reproductive cycle of yaks (72). The average length of the estrus cycle in yaks is 20 days, 1 day less than cattle. The signs of the estrus in yaks are not usually clear as in cattle (73). The duration of the gestation period of a yak is lower than that of a cow and lasts for 258 days. The stillbirth and abortion rates of yaks are between 5 and 10%. Normally, bulls mature at the age of 3–4 years old but they can breed properly at the age of 6–7 years old (72). Different authors suggested that maturity, conception rate, and other reproductive traits can be improved by using proper management techniques (5). The breeding season of yaks is affected by seasonal changes and other factors, such as environment, shortage of grass, latitude, and altitude (5, 74, 75). In the summer season, when the increase in temperature and humidity lead to a higher grass growth, yaks improve their body weight, particularly the females, and start their breeding season. Yaks' breeding season is at its peak from July to August as the temperature of the environment is higher compared to other months (72). In addition, this increase in temperature also improves the semen quality of yaks and the highest quality, quantity, and motility of the season observed from June to September and these semen parameters are also affected with time (72). Fertility depends on the number of services rather than the age of the bull. During the cold season, yaks increase their metabolism to maintain body temperature. During food scarcity and unavailability of forage due to cold harsh weather, yaks meet their nutritional requirement by using stored reserve and reducing body weight and body condition score by 17–25% (46).

There is a paucity of data on yak reproduction in the wild. Studies on domestic and wild yaks crossbreeding concentrated on male reproduction, particularly semen traits and hybrid productivity, and make up the majority of the data now available. For thawed and fresh semen, the motility of wild yaks is generally 63 and 39%, respectively (76). At 0 to 4°C, fresh semen can survive for 57 h when diluted with 7% glucose. Semen that has been frozen and then thawed at 37°C survives for 12 h (77). The resistance coefficients of the sperm from domestic yaks, domestic cattle, and wild yaks are 144.000, 12.750, and 6.000, respectively (77). This data demonstrates the exuberant motility of wild yak sperm. Spermatozoa from yaks typically have flagella. The head and centerpieces of yak sperm from wild and domestic animals are similar in length and width, but the main portion is noticeably longer in wild yaks (78). In regions where yaks procreate, the first estrus in females typically happens between the second and fourth warm season after birth, or between the ages of 13 and 36 months. Since yaks are seasonal breeders with a high natural conception rate, the fertility of a yak herd is primarily influenced by the percentage of yak cows that return to estrus in the year of calving. Research conducted at Sichuan revealed that 78% of female herds became pregnant following their first service, 15% following the second, and 7% following the third or more (79). Moreover, puberty onset is significantly influenced by nutritional conditions and the rate of live weight increase, with faster-growing yak heifers experiencing it earlier (79).

## 9. Milk composition of yaks

Yaks play an important role in the production of meat, milk, fur, and cheese for the people of the QTP (80). As mentioned earlier, the QTP has low temperatures, high altitudes, and low precipitation, and these aspects directly influence plant productivity and their nutritive values (81). Normally, the duration for growth of grass is ~100–150 days, and the dormant period lasts for ~7 months. Each year, grass begins to re-green in April, reaches its peak biomass in August, and withers in November. Yaks usually graze on natural grass all year without receiving additional feed. Due to the long cold season, QTP grassland is withered, and the most serious condition is a heavy snow disaster, in which all the green fodder is covered with heavy thick snow, impeding yaks from accessing any grazing fodder. This causes dramatic loss in the body weight of yaks and mortality. Earlier studies revealed that once food is accessible again in the short-term in the warm season, yaks enter a compensatory growth condition, with better growth rates and feed efficiency (82). With evolution, yaks developed unique physiological traits to acclimatize to extremely high-altitude environments.

It is important to comprehend the genetic makeup of yaks and how it can affect milk biosynthesis because yaks serve as the region's main supply of milk for human consumption. The milk of yaks is highly nutritive compared to dairy cow milk, particularly in protein and fat contents (83). The fat percentage in yak milk ranges from 5.5 to 7.5% as compared to 3.5% in bovine milk and the protein percentage ranges from 4.9 to 5.9% compared to 3.14% in bovine milk (Table 2) (87). The seasonal change in feed, particularly during the cold season, highly affected the traditional farming of yaks (87). Despite pastures' low protein content, using various supplements is not a usual practice (88). Consequently, determining yak milk composition, influenced by fodder grown at diverse phenological periods, is important to hypothetically increase milk quality and production.

**Table 2:** Yak milk composition.

**Item**	**Milk composition (g/100 g fresh Milk)**
Fat	6.12 ± 0.60
Total Protein	4.95 ± 0.53
Lactose	5.03 ± 0.43
Ash	0.79 ± 0.05
Total Solid	16.88 ± 1.36
	**(mg/kg)**
Cu	1.07 ± 0.08
Mg	154.10 ± 13.22
Zn	7.31 ± 0.44
Fe	0.57 ± 0.04
Mn	0.06 ± 0.01
Ca	1,545.45 ± 145.61
P	922.04 ± 70.13

Li et al. (84), Fox et al. (85), and Fox (86).

When milked during the main lactating period, yak milk is a naturally concentrated milk due to the greater levels of fat (5.7–7.5%), protein (4.0–5.9%), and lactose (4.0–5.9%) (19). The milk and different milk products made from yak milk are the major ingredients in the everyday life of Tibetan herders, and in areas where yaks graze on alpine meadows and mountain pastures, especially for the weak, elderly, ill, and young people of the region (19). Yak milk and various products made from yak milk, such as butter and cheese, are important sources of various vitamins and are critical sources of food for the people of the Tibetan Plateau due to the lack of vegetables, fruits, and the few resources of food (19).

However, the low volume and seasonal cyclicity of individual milk production, of roughly 150–500 kg of fresh milk per lactation, limit the large-scale industrialization of yak milk production. These factors include yak breed, parity, age, body condition, raising areas, pasture quality, pasture growth, milking techniques, milking time, and other environmental factors (84).

Different milk products made from yak milk garner interest due to their special nutritional composition. Many companies in China provide fresh pasteurized and UHT-treated milk. The chemical composition and chemical properties of yak milk are important from an economical point of view, as is the successful establishment of an yak products industry and marketing. The chemical composition of yak milk is different than that of cow milk, as the milk of yak is mainly produced by seasonal breeding. The yak milk composition depends on the availability of seasonal grass and also environmental conditions (89). During the mid-lactation period in yaks, solids like lactose, protein, and amino acids was found to be higher, while the fat content in yak milk continuously increased during the late lactation period (89). In contrast, milk from cow herd differs little with the change of seasons due to cows' year-round breeding, meaning that cow milk composition displayed minimum changes throughout the year (90).

In China, the milk composition of the Maiwa yak breed was studied by Sheng et al. (90), and the researchers focused on the mineral content, nitrogen distribution, and chemical composition of milk in the QTP, China. Furthermore, the composition of yak milk in the cold and warm seasons was established by the researchers, as mentioned in Table 2 (91, 92).

## 10. Yak meat

In the QTP region, yaks play a vital role in the living standard of the people due to their production, which contributes to the economy. Yaks provide milk, milk products, meat, and meat products as food items; fur and hide for the textile industry for leather and cloths; and manure for use as soil fertilizer and fuel (93). Normally, in the traditional system, yaks are domesticated and grazed on natural pasture without any supplement feeding. Therefore, in the winter season, the nutritional value of the pasture is reduced, which alternatively affected the growth rate of yaks (94). Long et al. (24) reported that in the cold season, due to low nutritional value in grazing pasture, yaks lose ~25% of their body weight and decrease their growth performance, which harms their economic effectiveness. Due to this situation, the management of yak domestication has changed in recent years. It was decided that a portion of finishing yaks would progressively transition from traditional grazing to semi-housing and housing management, which improved productivity and increased profit. Even though earlier studies found that yaks' nutritional requirements and metabolism differed from those of lowland ruminants, no feeding strategy has been developed to date to help fatten yaks. Consequently, yak feedlots have different feeding plans, and the results of yak fattening are not as positive as they are for cattle steers.

Regarding the quality of yak meat, the fat percentage is lower but the protein percentage is higher and with abundant vital amino acids, fatty acids, and minerals than commercial beef meat from the lowland region (95, 96). In the Tibetan region, the main source of protein in people's diet, ~50%, originates from yak meat (97, 98). However, there is still a significant discrepancy between the high demand for yak meat consumption and the inefficient output in the yak sector. The average daily gain and meat redness were upsurged when yaks received rumen-protected amino acid in their diets, while lightness, yellowness, drip loss, and tenderness were decreased, and these auspicious effects were revealed by the rumen-protected lysine and methionine supplementation in the yak diet. The supplementation of the rumen-protected amino acid in the diet of yaks increased the growth performance and meat quality, and for each fattening yak, the recommended doses of rumen-bypassed methionine and lysine should be given at rates of 15.0 and 5.0 g per day, respectively.

In meat quality, the fat and fatty acids profile plays a crucial role, and these parameters affect the appearance, texture, flavor, and hardness of the meat (99–101), as well as play a crucial part in preventing dark cutting, drip loss, and cold shortening (102). Optimum marbling in the meat is important in animal husbandry (103, 104). Demand, supply, and acceptability of unusual and exotic meats are rising globally along with the quickening pace of globalization and a shift in consumer philosophy (105, 106). Presently, the yak population is about 14 million worldwide. People living in the QTP region use the meat of yaks as a major source of animal protein (107, 108). Due to its lower fat content and higher protein content, devoid of anabolic steroids and pollutants, yak meat is gradually popular with consumers (109). The production of yak meat is more than 500 tons annually. The productions of carcass and dressing percentage of yak are 46.67–51.0 and 37.10–43.02%, respectively.

Yak meat, particularly the fatty acids in the meat, can be influenced by various factors, such as genetic variables (110), nutrition (111), gender (112), breeding environment (113), and etc. The metabolic responses are different in male and female animals (114), and their different hormones affect their fat composition (115).

## 11. Conclusion

In conclusion, domestication of yaks in the QTP plays an essential role to provide meat, milk, leather, fur, manure, etc. to local people and, due to these benefits, yaks are gaining importance worldwide. However, the high altitude, low temperature, low oxygen level, and harsh environment of the QTP subsequently affect the growth performance (growth-retarded yaks) and the survival of yaks. Yaks have a unique rumen ecology by hosting an essential gastrointestinal microbial community for pasture, fodders, and roughage degradation, yielding a high concentration of volatile fatty acids and microbial protein synthesis required for their maintenance and productivity. Information related to factors that influence the life of yaks and the microbial community demonstrated the novel rational avenues for designing strategies aimed at improving animal welfare and enhancing economic value. Furthermore, omics studies, particularly metabolomics and proteomics studies, should be carried out to provide additional information as to how these environmental factors influence yaks' survival and productive efficiency.

## Author contributions

Conceptualization: AS and MW. Writing—original draft preparation: AS. Writing—review and editing: AS, IB, IQ, MM, and MW. Visualization and supervision: MW. All authors have read and agreed to the published version of the manuscript.
